# Hypoxia-Driven M2-Polarized Macrophages Facilitate Cancer Aggressiveness and Temozolomide Resistance in Glioblastoma

**DOI:** 10.1155/2022/1614336

**Published:** 2022-08-22

**Authors:** Ge Zhang, Xiang Tao, Baowei Ji, Jie Gong

**Affiliations:** ^1^Department of Neurosurgery, Renmin Hospital of Wuhan University, Wuhan, 430060 Hubei, China; ^2^Department of Neurosurgery, General Hospital, Central Theater Command, PLA, Wuhan, 430070 Hubei, China

## Abstract

Hypoxia-induced M2 phenotypes of tumor associated macrophages (TAMs) promote the development and chemoresistance of multiple types of cancers, including glioblastoma (GBM). However, the detailed molecular mechanisms have not been fully understood. In this study, we firstly reported that hypoxic pressure promoted M2 macrophage generation, which further promoted cancer progression and temozolomide (TMZ) resistance in GBM through secreting vascular endothelial growth factor (VEGF). Specifically, the clinical data suggested that M2 macrophages were significantly enriched in GBM tissues compared with the adjacent normal tissues, and the following *in vitro* experiments validated that hypoxic pressure promoted M2-polarized macrophages through upregulating hypoxia-inducible factor-1*α* (HIF-1*α*). In addition, hypoxic M2 macrophages VEGF-dependently promoted cell proliferation, epithelial-mesenchymal transition (EMT), glioblastoma stem cell (GSC) properties, and TMZ resistance in GBM cells through activating the PI3K/Akt/Nrf2 pathway. Also, M2 macrophages secreted VEGF to accelerate angiogenesis in human umbilical vein endothelial cells (HUVECs) through interacting with its receptor VEGFR. In general, we concluded that hypoxic M2 macrophages contributed to cancer progression, stemness, drug resistance, and angiogenesis in GBM through secreting VEGF, and our data supported the notion that targeting hypoxia-associated M2 macrophages might be an effective treatment strategy for GBM in clinical practices.

## 1. Introduction

Glioblastoma (GBM) is the most common and lethal malignancy in brain, and according to the latest data, the 2-year survival rate for this disease is below 10%, and GBM seriously degrades the life quality of human beings worldwide [1, 2]. However, the initiation, pathogenesis, and progression of GBM are very complicated, and researchers agree that the tampered tumor immune microenvironment (TIME) plays critical role to accelerate the development of GBM [3–5]. Mechanistically, to evade immune surveillance, cancer cells interact with the infiltrating lymphocytes and transform those immune cells from antitumor status into protumor status [3–5]. For example, cancer cells derived programmed ligand 1 (PL-L1) induce T lymphocyte death through binding to its receptor PD-1 [5, 6]. Among all the cancer-associated lymphocytes, tumor-associated macrophages (TAMs) are closely related with GBM progression and prognosis, and TAMs can be functionally divided into two subtypes, including M1- and M2-polarized macrophages [7–9]. Of note, M2 macrophages but not M1 subtypes promote GBM aggressiveness, and blockage of M2 polarization in TAMs is effective to restrain GBM development [10–12]. Based on the existed information [13, 14], M2 macrophages communicate with the surrounding cells through secreting various molecules, such as EGF, PDGF, VEGF, and TGF-*β*1, which are all identified as pivotal contributors for cancer aggressiveness [15, 16], but it is still unclear whether M2 macrophages facilitate GBM progression through secreting those factors.

Temozolomide (TMZ) is commonly used for the treatment of GBM in clinic, but TMZ resistance has become a big obstacle that makes this chemical drug ineffectiveness [17, 18]. Thus, it is urgent and necessary to search for the novel strategies to improve TMZ sensitivity in GBM. As previously reported, glioblastoma stem cells (GSCs) and tumor-promoting microenvironment are two pivotal factors that contribute to TMZ resistance in GBM [19, 20]. Specifically, GSCs are identified as a subgroup of cells with self-renewal abilities, which also interact with the surrounding cells through delivering exosomes. Under the TMZ stresses, GSCs are forced to differentiate into the cancer cells with TMZ-resistant properties, and expansion of these GBM cells will make GBM cells resistant to further TMZ treatment [19, 20]. In addition, GBM cells are capable of sustaining a tumor-promoting microenvironment to antagonize TMZ treatment, and interestingly, it has been reported that M2-polarized macrophages contribute to TMZ resistance in GBM [21, 22]. For example, Chuang et al. report that elimination of M2 macrophage-derived miR-21 exosomes overcomes TMZ resistance in GBM [22]. CD163 is a typical surface biomarker for M2 macrophages, and Miyazaki et al. find that CD163-positive M2 macrophages also increase resistance of immune therapy in TMZ-resistant GBM cells [21]. Also, as previously described, M2-polarized macrophages also promote cancer stemness in pancreatic cancer [23] and thyroid cancer [24]. Thus, it is reasonable to speculate that M2 macrophages may contribute to cancer stemness and TMZ resistance in GBM, but the detailed mechanisms are still needed to be elucidated.

Hypoxic condition is common phenomena in the solid tumors with volumes above 2 mm^3^, and hypoxia has been reported to regulate both TIME and chemoresistance in multiple cancers [25, 26]. For example, Park et al. illustrate that hypoxia-induced tumor exosomes promote M2-like macrophage polarization [27], Qi et al. report that hypoxia correlates with poor survival and M2 macrophage enrichment in colorectal cancer [28], Ge et al. find that hypoxia-mediated inhibition of mitochondria apoptosis promotes TMZ resistance in GBM, and Dico et al. verify that hypoxic pressure serves as a switch for GBM responsiveness to TMZ [29]. In addition, hypoxia is found to promote cancer stem cell properties in GBM [30, 31]. Nevertheless, the detailed molecular mechanisms by which hypoxia promotes M2 macrophage polarization, TMZ resistance, and stemness in GBM have not been fully delineated. As the results of oxygen deprivation, the characteristics of the endothelial cells and stromal cells were significantly changed, which are forced to form tumor vessels and facilitate tumor angiogenesis [32–34]. Those tumor vessels will ameliorate hypoxic conditions, and on the other hand, they facilitate cancer metastasis [32–34]. Interestingly, M2 macrophages are found to facilitate cancer angiogenesis [35, 36], but the detailed molecular mechanisms are still unclear.

Thus, the present study focused on investigating the role and molecular mechanisms by which hypoxia-induced M2 macrophages contributed to the development and TMZ resistance of GBM, which will provide alternative treatment strategies for GBM in clinical practices.

## 2. Materials and Methods

### 2.1. Collection and Analysis of the Clinical Specimens

The patients diagnosed as GBM (*N* = 42) were recruited, and the cancerous and adjacent normal nonmalignant tissues were obtained by surgery. According to their treatment history, the recurrent GBM patients (*N* = 15) with TMZ treatment history were judged as TMZ-resistant GBM, and the rest of the patients (*N* = 27) without chemotherapy history were deemed as TMZ-sensitive GBM. All the clinical experiments were conducted followed the tenets of the Declaration of Helsinki and were approved by the Ethics Committee of Renmin Hospital of Wuhan University. We had obtained the signed inform consent forms from all the participants.

### 2.2. Cell Culture and Vector Transfection

The GBM cell lines, including LN229 and U251, and the THP-1 cells were purchased from Shanghai Cell Biology Institute of Chinese Academy of Sciences (Shanghai, China), which were cultured in the DMEM with 10% fetal bovine serum supplementation. To conduct the TMZ-resistant experiments, the GBM cells were subjected to high-dose TMZ treatment (1200 *μ*M) as previously reported. In addition, according to the previous publications [37–39], the THP-1 cells were treated with phorbol 12-myristate 13-acetate (PMA) (20 nM for 24 h) to differentiate into the CD68-positive M0 macrophages, which were further subjected to hypoxic conditions for 12 h to induce M2 macrophages. The shRNAs for VEGF, EGF, PDGF, TGF-*β*1, VEGFR, and Nrf2 were designed by Sangon Biotechnology (Shanghai, China) and were delivered into the M2 macrophages by using the Lipofectamine 2000 transfection kit purchased from Invitrogen (CA, USA).

### 2.3. Real-Time qPCR Analysis

Total RNA was extracted by TRIzol reagent (Invitrogen, USA), and Real-Time qPCR analysis was performed to examine the mRNA levels of CD206, CD80, HIF-1*α*, CD163, CDK2, CDK6, Cyclin D1, N-cadherin, Slug, Twist, E-cadherin, CD133, OCT4, and Nanog according to the standard experimental procedures documented in the previous literatures [10].

### 2.4. Western Blot Analysis

RIPA lysis buffer (Beyotime, Shanghai, China) was used to extract total proteins, which were further separated by 10% SDS-PAGE based on their molecular weight. The proteins were then transferred onto the PVDF membranes (Millipore, USA) and blocked by 5% nonfat milk. The membranes were subsequently incubated with the primary antibodies against CD68 (Abcam, UK), HIF-1*α* (Abcam, UK), CD206 (Abcam, UK), CD163 (Abcam, UK), CD80 (Abcam, UK), CD133 (Abcam, UK), p-PI3K (Abcam, UK), PI3K (Abcam, UK), p-Akt (Abcam, UK), Akt (Abcam, UK), and GAPDH (Abcam, UK) at 4°C overnight, which were further subjected to the secondary antibodies, and the ECL system (BioRad, USA) was employed to visualize the protein bands, and the grey values were calculated by using the Image J software (National Institutes of Health, USA), which represented the relative expression levels of the associated genes.

### 2.5. Immunofluorescence Staining Experiment

The GBM cells were cultured and blocked with 5 mg/ml BSA for 1 h, were subsequently incubated with the primary antibody against Nrf2 at 4°C overnight, and then stained with the fluor 555-conjugated secondary antibody (1 : 500) for 1 h at room temperature. Then, the cells were washed by PBS buffer and restained with DAPI for nucleus. An Immunofluorescence Detection System (BioGeneX, CA, USA) was employed to observe and capture the immunofluorescent signals, and the fluorescent intensity was analyzed by performing the Image J software (National Institutes of Health, USA).

### 2.6. Enzyme-Linked Immunosorbent Assay (ELISA)

The commercial ELISA kits (Cell Signaling Technology, USA) were purchased to examine the expression levels of VEGF, EGF, PDGF, and TGF-*β*1 in the cells' supernatants and the conditional medium according to the protocols provided by the producers. The absorbance values were measured and analyzed by using the ELISA microplate spectrophotometer (Biotek, USA) at the wavelength of 450 nm and 550 nm.

### 2.7. 3-(4,5)-Dimethylthiahiazo-(-z-yl)-3,5-Diphenytetrazoliumromide (MTT) Assay

The GBM cells were cultured in the 96-well plates at the density of 1,000 cells per well, and the MTT reaction solution with 20 *μ*l/well was added to the cells for 4 h at 37°C. Then, the cell supernatants were discarded, and the formazan was dissolved by 150 *μ*l of DMSO in each well. The plates were fully vortexed, and a microplate reader (BioRad, USA) was used to examine the optical density values, which represented relative cell proliferation abilities.

### 2.8. Examination of Cell Apoptosis

The GBM cells were sequentially stained with Annexin V-FITC and PI according to the instructions of the commercial Apoptosis Detection kit (YEASEN Biotech, Shanghai, China), and a flow cytometer (FCM, BD Bioscience, USA) was employed to examine the Annexin V-FITC and PI-positive apoptotic cell ratio.

### 2.9. Tube Formation Assay

The 96-well plates were coated with the precooled growth factor-free Matrigel (BD Bioscience, USA) with 50 *μ*l each well for 30 min, and the human umbilical vein endothelial cells (HUVECs) with serum deprivation were seeded onto the gel at the concentration of 3 × 10^4^ each well, and 200 *μ*l of the M2-CM and normal medium was added to the wells. The cells were cultured for 12 h, and the tube formation of the cells was observed under a light microscope (ThermoFisher Scientific, USA).

### 2.10. *In Vivo* Tumor-Bearing Mouse Models

The U251 cells were diluted in the PBS buffer and were subcutaneously injected into the dorsal flank of the male BALB/c mice (4-6 weeks old) at the concentration of 5 × 10^5^ cells per mouse, which were further administered with M0-CM and M2-CM coinjection. At about 25 days postinjection, the mice were anesthetized by 100 mg/mg Barbiturate, and the tumors were obtained by surgery and the tumor weights were calculated. All the animal experiments were approved by the Ethics Committee of Renmin Hospital of Wuhan University.

### 2.11. Collection and Analysis of the Data

Data were presented as means ± standard deviation (SD), and data analysis was performed by using the SPSS 18.0 software and GraphPad Prism 8.0 software. Comparisons between two groups were conducted by using the Student *t*-test, and data from multiple groups were analyzed by using the one-way ANOVA analysis. Kaplan-Meier survival analysis was used to determine the correlations of M1/M2 macrophages with GBM patients' prognosis. ^∗^*P* < 0.05 was regarded as statistical significance.

## 3. Results

### 3.1. Association of M2 Macrophages with GBM Aggressiveness, Prognosis, and Clinical Characteristics

**Figure 1:**
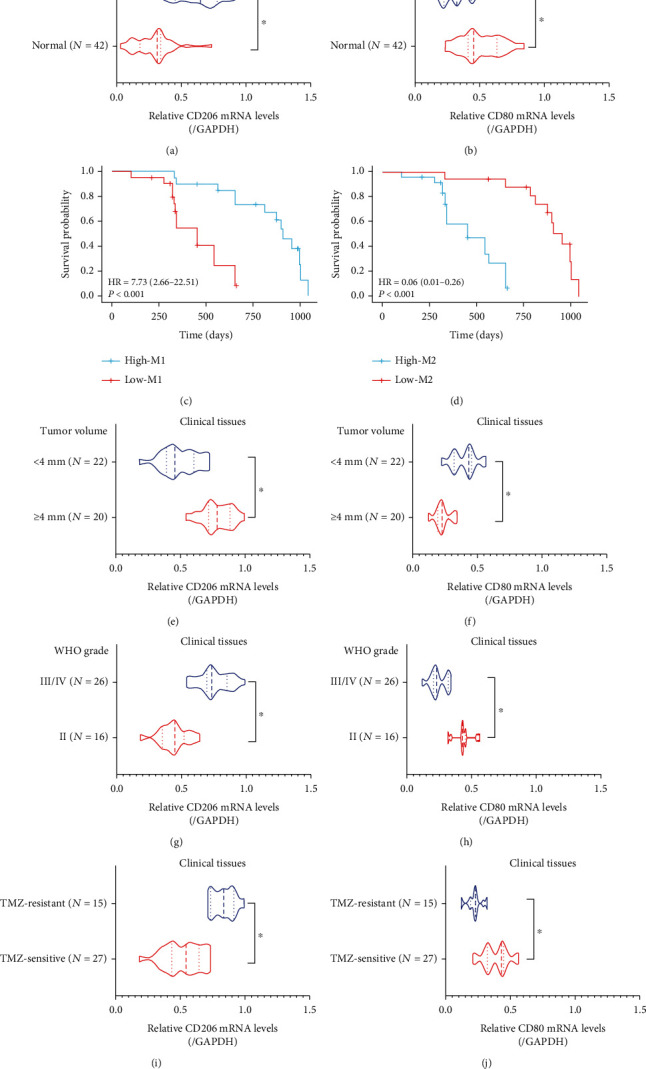
M1/M2 macrophages were closely related with GBM progression, prognosis, and drug resistance in clinic. (a, b) The expression levels of CD206 and CD80 in GBM tissues were examined by Real-Time qPCR analysis. (c, d) Kaplan-Meier survival analysis was performed to analyze the correlation of M1/M2 macrophages with patients' prognosis. The correlations of M1/M2 macrophages with (e, f) tumor volume and (g, h) WHO grade were analyzed. (i, j) The relevance of M1/M2 macrophages with TMZ resistance in the clinical GBM tissues was analyzed by performing Real-Time qPCR analysis. Each experiment repeated at least 3 times, and ^∗^*P* < 0.05.

Infiltrating TAMs are critical components to constitute the tumor-promoting microenvironment in tumors, and previous literatures agree that M2-polarized macrophages act as important contributors for the development of GBM [10–12], which were verified by our experiments. The clinical samples of GBM tissues were initially collected, and the biomarkers of M2 macrophage (CD206) and M1 macrophage (CD80) were examined by performing Real-Time qPCR analysis, which showed that the mRNA levels of CD206 were increased (Figure 1(a)), whereas CD80 were decreased (Figure 1(b)) in the GBM tissues compared to the adjacent normal tissues. Next, we analyzed the correlations of TAMs with patients' prognosis and expectedly found that patients with better prognosis and longer survival time were prone to have lower ratio of M2 macrophages but higher ratio of M1 macrophage (Figures 1(c) and 1(d)), hinting that the M1 and M2 macrophages were pivotal indicators for GBM patients' prognosis. Then, we analyzed the correlations of M2 macrophage with the clinical characteristics of GBM patients and found that the M2 macrophage signature (CD206) was significantly upregulated, whereas M1 marker CD80 was downregulated in GBM patients with bigger tumor volume (>4 cm) (Figures 1(e) and 1(f)) and higher WHO grade (WHO III/IV) (Figures 1(g) and 1(h)), but their expressions had nothing to do with patients' gender (Supplementary Figure S1A, B) and age (Supplementary Figure S1C, D), indicating that the enrichment of M1 and M2 macrophages was relevant to GBM progression. Previous literatures describe that M2-polarized macrophages also contribute to drug resistance [21, 22], and we verified that CD206 was significantly upregulated, and CD80 was downregulated in the cancer tissues collected form recurrent GBM patients suffered from TMZ treatment, in contrast with the patients without TMZ treatment (Figures 1(i) and 1(j)), which supported the notion that enrichment of M2 macrophages was relevant to TMZ resistance in GBM.

### 3.2. THP-1-Derived Macrophages Were Prone to Form M2-Polarized Subtypes under Hypoxic Stresses

**Figure 2:**
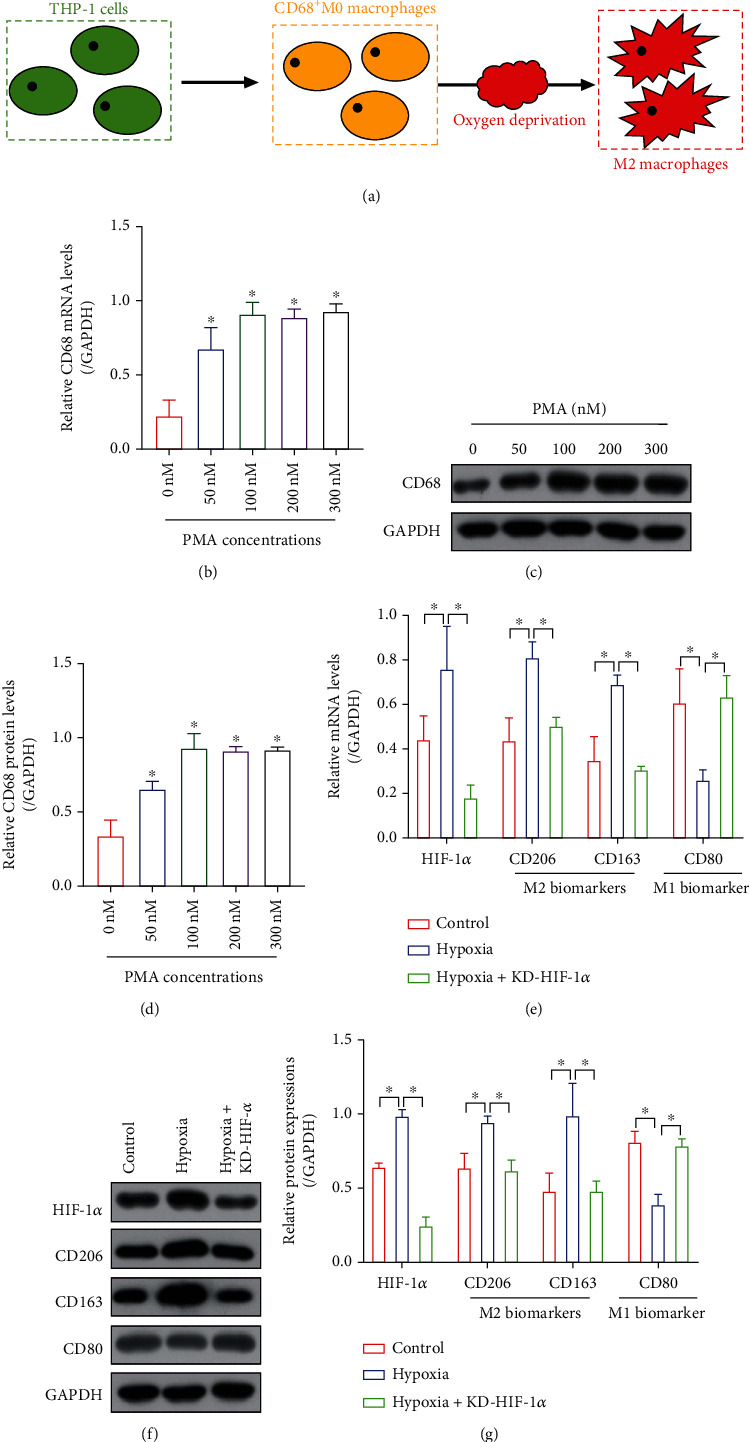
Hypoxic conditions promoted M0-to-M2 transition in the THP-1 cells. (a) The graphical illustration for the induction of M2 macrophages from THP-1 cells. PMA promoted the generation of CD68-positive M0 macrophages in a dose-dependent manner, as it was, respectively, determined by performing (b) Real-Time qPCR and (c, d) Western Blot analysis. (e) Real-Time qPCR and (g, g) Western Blot analysis verified that oxygen deprivation promoted M2 macrophage generation through inducing the upregulation of HIF-1*α*. Each experiment repeated at least 3 times, and ^∗^*P* < 0.05.

Hypoxia happens in solid tumors when tumor volume reaches above 2 mm^3^, and oxygen deprivation exerts pressure and alters the biological characteristics of both tumor cells and surrounding cancer-associated stroma/immune cells, which facilitates the formation of tumor-promoting circumstances [25, 26]. To ask whether hypoxic conditions change M1/M2 polarization, in our experiments, the THP-1 cells were treated with phorbol 12-myristate 13-acetate (PMA) to differentiate into the CD68-positive M0 macrophages (Figure 2(a)). The results showed that PMA treatments upregulated the pan-macrophage biomarker CD68 in the THP-1 cells in a dose-dependent manner at both mRNA (Figure 2(b)) and protein (Figures 2(c) and 2(d)) levels. The PMA-induced M0 macrophages were further subjected to oxygen deprivation (Figure 2(a)), and we performed the Real-Time qPCR and Western Blot analysis to analyze the expression status of M1/M2 macrophage-associated biomarkers (Figures 2(e)–2(g)). Interestingly, the results showed that hypoxic stimulations upregulated CD206 and CD163 to promote M2 polarization but suppressed the expression levels of M1-associated biomarker (CD80) (Figures 2(e)–2(g)), suggesting that hypoxic stresses promoted M0-to-M2 macrophage transition. Next, the potential underlying mechanisms by which hypoxia-induced M2 macrophage were determined. As previously reported, HIF-1*α* is a critical gene that can be induced by hypoxic conditions [40], which is closely related with the polarization of M2 macrophage [41, 42]. In our experiments, we verified that hypoxia upregulated HIF-1*α* in M0 macrophages, and silencing of HIF-1*α* abrogated the promoting effects of hypoxic stimulation on M2 macrophage polarization (Figures 2(e)–2(g)). Collectively, the above data hinted that hypoxia promoted M2 macrophage generation in a HIF-1*α*-dependent manner.

### 3.3. Hypoxic M2 Macrophages Secreted VEGF to Accelerate Cancer Aggressiveness and Angiogenesis in GBM

**Figure 3:**
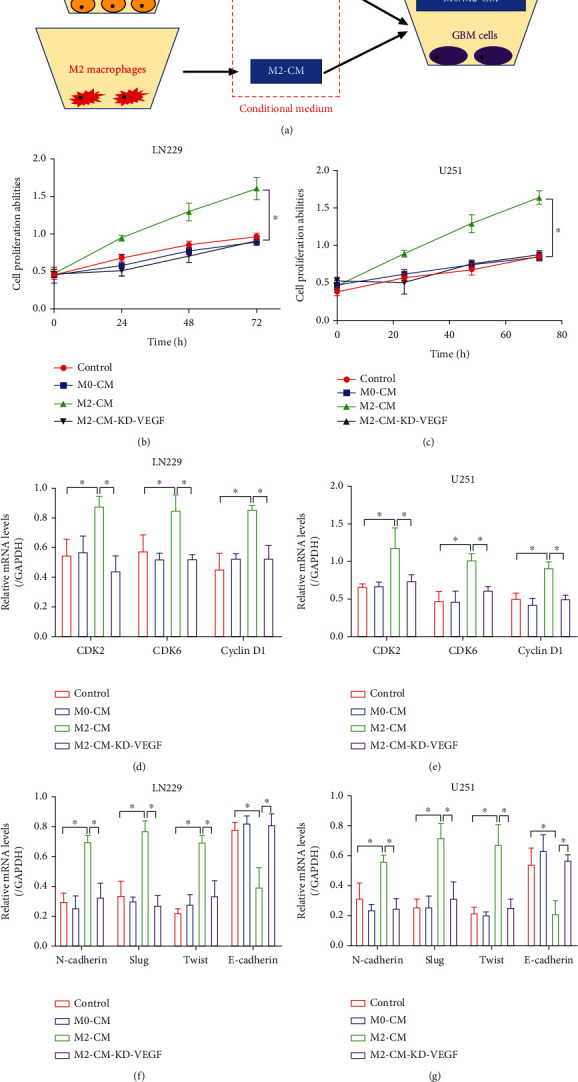
Hypoxic M2 macrophage-derived conditional medium facilitates GBM aggressiveness and angiogenesis in a VEGF-dependent manner. (a) The graphical illustration for the coculture of GBM cells and the macrophage-derived conditional medium. (b, c) GBM cell proliferation abilities were determined by performing MTT assay. (d, e) The cell mitosis-associated biomarkers (CDK2, CDK6, and Cyclin D1) were examined by performing Real-Time qPCR analysis. (f, g) The EMT-associated signatures (N-cadherin, Slug, Twist, and E-cadherin) were examined by Real-Time qPCR analysis. Each experiment repeated at least 3 times, and ^∗^*P* < 0.05.

Interactions between cancer cells and M2 macrophages are pivotal for accelerating the aggravation of multiple cancers, which sustain the tumor-promoting microenvironment in solid tumors [25, 26]. Thus, we next explored whether hypoxia-induced M2 macrophage contributed to cancer progression in GBM. To achieve this, the conditional medium of THP-1 with or without hypoxic stimulation was incubated with the GBM cell line LN229 and U251 (Figure 3(a)), and our MTT assay results suggested that hypoxic M2 macrophage conditional medium (M2-CM) significantly increased the proliferation abilities in the GBM cells (Figures 3(b) and 3(c)), which were supported by the Real-Time qPCR analysis results that M2-CM also increased the mRNA levels of CDK2, CDK6, and Cyclin D1 to promote cell mitosis in the GBM cells (Figures 3(d) and 3(e)). Also, our results showed that M2-CM increased the expression levels of mesenchymal biomarkers (N-cadherin, Slug, and Twist), whereas downregulated epithelial marker (E-cadherin) to promote epithelial-mesenchymal transition (EMT) in GBM cells (Figures 3(f) and 3(g)). Furthermore, the xenograft tumor-bearing mouse models were established by using the U251 with or without M2-CM coincubation, and the results showed that M2-CM promoted tumor growth *in vivo* (Supplementary Figure S3A, B). According to the previous studies [15, 16], M2 macrophages interact with the surrounding cells through secreting the growth factors including EGF, PDGF, VEGF, and TGF-*β*1, which are considered to be closely associated cancer progression. The above information encouraged us to explore by which growth factors that mediated M2 macrophage-induced cancer aggressiveness in GBM. To achieve this, the shRNAs for EGF, PDGF, VEGF, and TGF-*β*1 were delivered into the hypoxic M2 macrophages (Supplementary Figure S2A-D), and we surprisingly found that deletion of VEGF, instead of other growth factors, significantly abrogated the promoting effects of M2-CM on cell proliferation (Figures 3(b) and 3(c), Supplementary Figure S2E-F). Also, knockdown of VEGF abrogated the promoting effects of M2-CM on GBM cell mitosis (Figures 3(d) and 3(e)), EMT (Figures 3(f) and 3(g)), and tumorigenesis (Supplementary Figure S3A, B) in GBM *in vitro* and *in vivo*. Angiogenesis is a critical process for cancer metastasis [32–34], and M2 macrophages have been reported to facilitate cancer angiogenesis [35, 36], which were verified by our results that M2-CM promoted tube formation abilities in HUVECs, which were abrogated by both silencing VEGF in the M2-CM and deletion of VEGFR in HUVECs (Supplementary Figure S4A, B), suggesting that M2 macrophages secreted VEGF, which interacted with VEGFR to activate its downstream signals and eventually facilitate tumor angiogenesis.

### 3.4. Hypoxia-Elicited M2 Macrophage Promoted Stemness and TMZ Resistance in GBM Cells by Secreting VEGF

**Figure 4:**
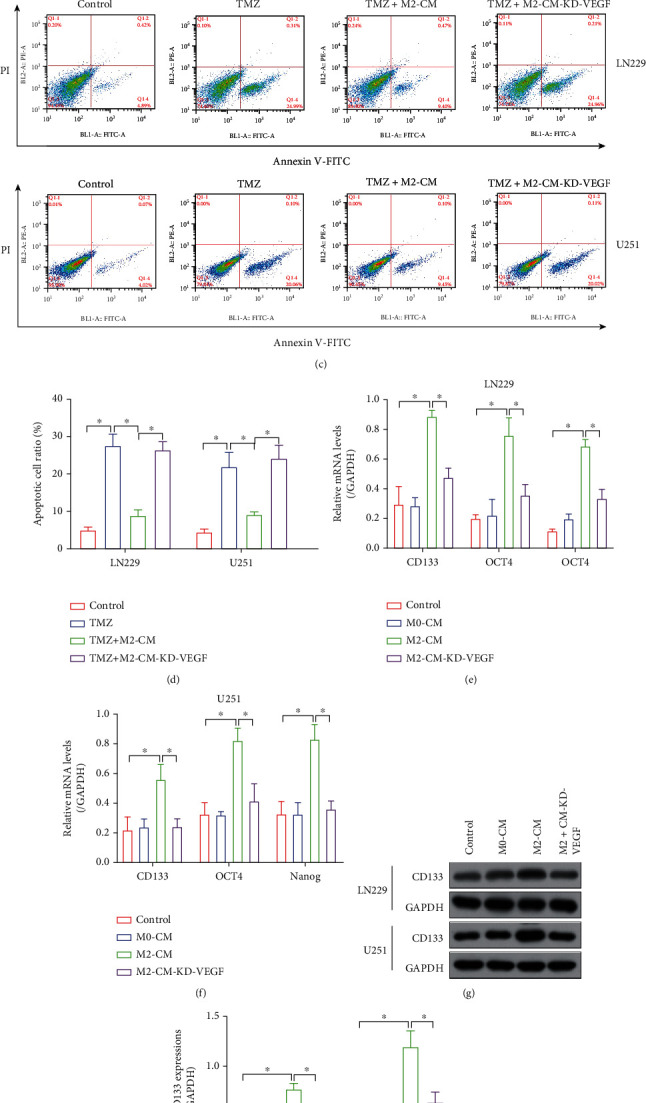
M2-CM VEGF-dependently increased TMZ resistance in the GBM cells. (a, b) MTT assay results verified that M2-CM increased cell proliferation in the GBM cells cotreated with TMZ. (c, d) FCM assay verified that M2-CM suppressed TMZ-induced apoptotic cell death in the GBM cells. (e, f) Real-Time qPCR and (g, h) Western Blot analysis were used to examine the expression status of GSC-related proteins (CD133, OCT4, and Nanog). Each experiment repeated at least 3 times, and ^∗^*P* < 0.05.

M2 macrophage VEGF-dependently contributes to GBM aggressiveness, and M2 macrophage is also involved in regulating chemoresistance in cancers. In addition, VEGF has been verified as a critical growth factor that increases drug resistance in multiple cancers [43, 44], and we next explored whether M2 macrophage increased TMZ resistance in GBM through VEGF. To this end, the hypoxic M2-CM with or without VEGF deletion was cocultured with the GBM cells, and the cells were subsequently subjected to TMZ treatments. The MTT assay results showed that hypoxic M2-CM significantly increased cell viability in TMZ-treated LN229 and U251 cells, which were abolished by knocking down VEGF in the M2-CM (Figures 4(a) and 4(b)). Similarly, the FCM results showed that TMZ tended to induce cell apoptosis in the GBM cells without M2-CM supplementation, and the suppressing effects of M2-CM on TMZ-induced apoptotic cell death in GBM cells were reversed by deleting VEGF (Figures 4(c) and 4(d)). In addition, GSCs are identified as a group of GBM cells with undifferentiated status, which differentiate into chemoresistant GBM cells under chemical drugs' pressure, thus contributing to drug resistance in GBM. Interestingly, recent publications notice that GSC properties can be modulated by M2 macrophage. Based on the above information, our data verified that M2-CM VEGF-dependently increased the expression levels of GSC-associated genes, including CD133, OCT4, and Nanog, in the GBM cells (Figures 4(e)–4(h)). The above data hinted that M2 macrophage secreted VEGF contributed to TMZ resistance and cancer stemness in GBM.

### 3.5. M2 Macrophages Secreted VEGF to Activate the PI3K/Akt/Nrf2 Pathway in GBM Cells

**Figure 5:**
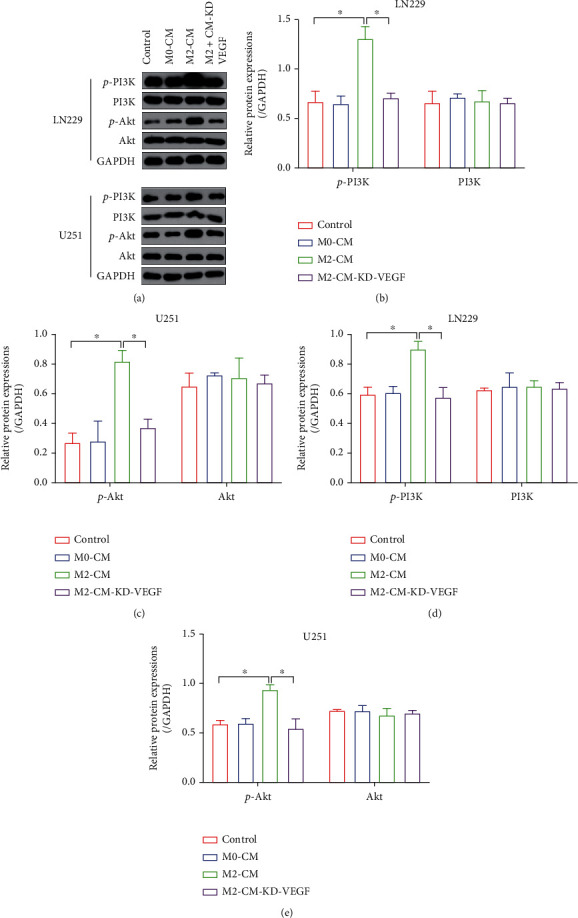
M2-CM activated the PI3K/Akt/Nrf2 pathway in a VEGF-dependent manner. (a–e) The expression levels of p-PI3K, PI3K, p-Akt, and Akt in the GBM cells were determined by performing Western Blot analysis. Each experiment repeated at least 3 times, and ^∗^*P* < 0.05.

According to recent literatures, VEGF exerts its biological functions through activating various downstream signaling pathways, including the classical PI3K/Akt/Nrf2 pathway, and activation of this signaling cascade facilitates cancer aggressiveness and drug resistance [45, 46]. Thus, we were wondering if M2 macrophage-derived VEGF activated the PI3K/Akt/Nrf2 pathway in GBM cells. To investigate this issue, the GBM cells were cocultured with M2-CM with or without VEGF deletion, and the Western Blot analysis results showed that M2-CM increased the expression levels of phosphorylated PI3K (p-PI3K) and Akt (p-Akt) in the GBM cells, which were suppressed by silencing VEGF (Figures 5(a)–5(e)). Also, the immunofluorescent staining assay was performed to examine Nrf2 expressions, and we expectedly found that M2-CM increased the expression levels of Nrf2 in the GBM cells in a VEGF-dependent manner (Supplementary Figure S5). Also, the promoting effects of M2-CM on Nrf2 expressions were abolished by cotreating cells with the PI3K inhibitor LY294002 (Supplementary Figure S5). Thus, we concluded that M2-CM-derived VEGF activated the PI3K/Akt/Nrf2 pathway in the GBM cells.

### 3.6. Targeting the PI3K/Akt/Nrf2 Pathway Abrogated the Promoting Effects of M2 Macrophages on GBM Progression, Stemness, and TMZ Resistance

**Figure 6:**
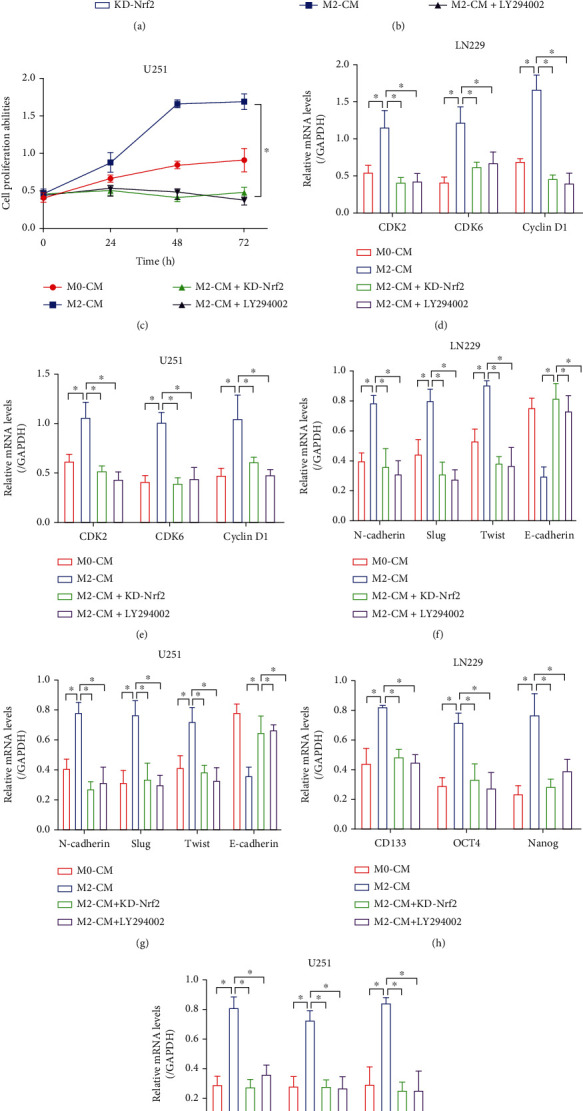
M2-CM promoted cancer aggressiveness through activating the PI3K/Akt/Nrf2 pathway. (a) The shRNA for Nrf2 was delivered into the GBM cells, and the transfection efficiency was determined by Real-Time qPCR. (b, c) Cell proliferation abilities were determined by performing MTT assay. (d, e) Real-Time qPCR was performed to determine the expression status of CDK2, CDK6, and Cyclin D1. (f, g) The EMT-associated markers were examined by conducting Real-Time qPCR. (h, i) The expression levels of the cancer stem cell biomarkers were determined by Real-Time qPCR analysis. Each experiment repeated at least 3 times, and ^∗^*P* < 0.05.

**Figure 7:**
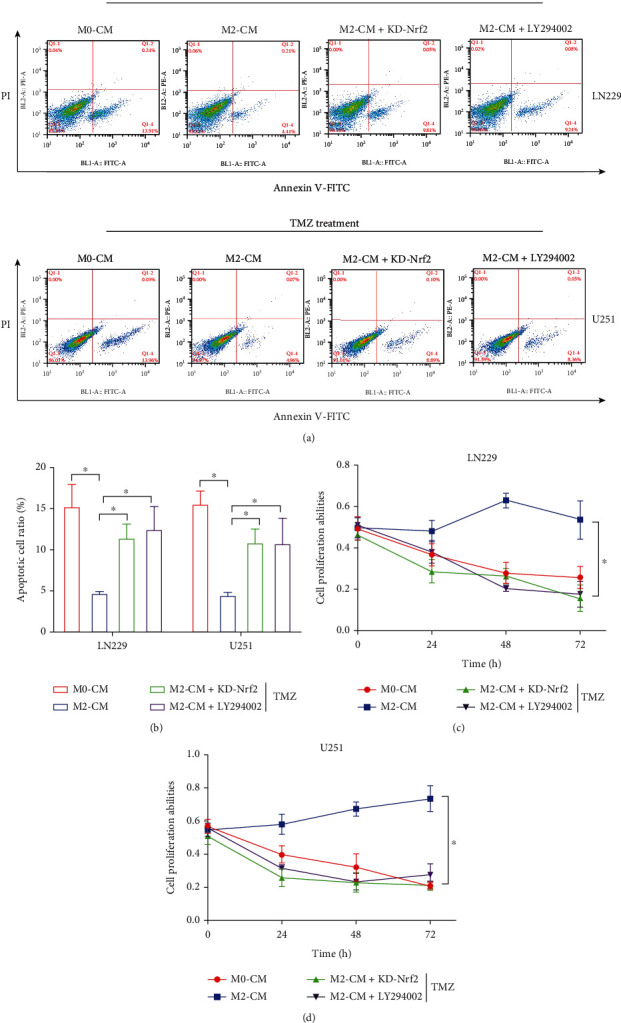
M2-CM increased the TMZ resistance in the GBM cells by regulating the PI3K/Akt/Nrf2 pathway. (a, b) The apoptotic cell ratio in the GBM cells was determined by performing the FCM assay. (c, d) The MTT assay was employed to determine cell proliferation in the GBM cells. Each experiment repeated at least 3 times, and ^∗^*P* < 0.05.

Nrf2 is known as the classical antioxidant gene [47], and TMZ exerts its cytotoxic effects on cancer cells partially depending on triggering excessive oxidative damages [48]. Also, activation of the PI3K/Akt pathway promotes TMZ resistance in multiple cancers [49, 50]. It was reasonable to speculate that M2 macrophage-derived VEGF promoted cancer aggressiveness and TMZ resistance in GBM through activating the PI3K/Akt/Nrf2 pathway. To verify this hypothesis, the hypoxic M2-CM-treated GBM cells were, respectively, administered with the PI3K inhibitor LY294002 and shRNA for Nrf2, and the following Real-Time qPCR results indicated that Nrf2 was successfully silenced in the GBM cells (Figure 6(a)). The MTT assay results showed that the promoting effects of M2-CM on cell proliferation in GBM cells were abrogated by both LY294002 and Nrf2 knockdown (Figures 6(b) and 6(c)). In addition, as it was determined by Real-Time qPCR, LY294002 and Nrf2 ablation also suppressed CDK2, CDK6, and Cyclin D1 expressions to block mitosis in the M2-CM-treated GBM cells (Figures 6(d) and 6(e)). Consistent with the above results, we noticed that M2-CM-induced GBM cell EMT (Figures 6(f) and 6(g)) was also suppressed by LY294002 and Nrf2 knockdown. Furthermore, as previously described, the PI3K/Akt/Nrf2 pathway regulates cancer stem cell properties, which were validated by our results that blockage of the PI3K/Akt/Nrf2 pathway by LY294002 and Nrf2 silence also suppressed GSC signatures (CD133, OCT4, and Nanog) (Figures 6(h) and 6(i)) in the GBM cells treated with M2-CM. Finally, to ask whether M2-CM influences TMZ resistance in a PI3K/Akt/Nrf2 pathway-dependent manner, we performed FCM assay and found that the rescuing effects of M2-CM on TMZ-induced GBM cell apoptosis were abolished by both LY294002 and Nrf2 knockdown (Figures 7(a) and 7(b)). The above results were supported by the following MTT assay that M2-CM rescued cell viability in TMZ-treated GBM cells, which were revered by LY294002 cotreatment and Nrf2 ablation (Figures 7(c) and 7(d)), suggesting that M2 macrophage-secreted VEGF to promote GBM aggressiveness, stemness, and TMZ resistance via activating the PI3K/Akt/Nrf2 pathway.

## 4. Discussion

In recent studies, hypoxia-associated M2 phenotypes of tumor-associated macrophages (TAMs) have been identified as crucial component of cancers, which contribute to the development of various types of cancer [51–53], including glioblastoma (GBM) [54], but the detailed molecular mechanisms have not been fully delineated. As previously reported, M2-polarized macrophages are closely related with the progression and prognosis in GBM patients [10–12], which were verified by our data that M2 macrophages are significantly enriched in the GBM tissues, and high proportion of M2 subtype macrophages predicted a worse prognosis in GBM patients. Although the involvement of M2 macrophages in regulating GBM development has been widely determined [10–12], the underlying mechanisms of M2 polarization are still unclear. Previous data suggest that hypoxic conditions in the solid tumor may be the reason [28]; thus, we discussed this issue and found that oxygen deprivation promoted M2 polarization in the M0 macrophage through upregulating HIF-1*α*. According to the existed information, HIF-1*α* is significantly upregulated under hypoxic stresses [40], and HIF-1*α* also participates in the regulation of M2 macrophage polarization [55, 56], which are in consistent with our results, suggesting that hypoxia promoted M2 macrophage generation in GBM through inducing HIF-1*α*. Then, we investigated the underlying mechanisms by which M2 macrophage promoted GBM aggressiveness, and we expectedly found that M2 macrophages VEGF-dependently promoted cell proliferation, epithelial-mesenchymal transition, tumorigenesis, and angiogenesis, which were supported by the existed data that VEGF itself serves as an oncogene in GBM [43, 44].

Temozolomide (TMZ) resistance is a huge obstacle which seriously limits the therapeutic effects of this chemical drug for the treatment of GBM [17, 18], and it becomes urgent to search for the novel strategies to increase TMZ sensitivity. Previous data suggest that M2 macrophages play important role in regulating chemoresistance [21, 22], which were validated by our experiments that M2 macrophage-derived conditional medium (M2-CM) significantly increased TMZ resistance in the GBM cells in a VEGF-dependent manner. As previously reported, upregulated VEGF levels are relevant to TMZ resistance [57, 58], which supported the notion that M2 macrophages promoted TMZ resistance in GBM through delivering VEGF. As one of the most important factors that contribute to chemoresistance, glioblastoma stem cells (GSCs) are also reported to be closely related with M2 macrophages [23, 24], and recent data hint that GSC properties can be modulated by VEGF [15, 45]. Hence, in our study, we validated that M2 macrophages secreted VEGF to promote cancer stemness in the GBM cells.

Then, we verified that M2 macrophage VEGF-dependently activated the PI3K/Akt/Nrf2 pathway to promote cancer aggressiveness and TMZ resistance in GBM. Based on the published data, the PI3K/Akt/Nrf2 pathway is identified as tumor-promoting signals that contribute to the development of various types of cancer, including GBM [59, 60], and this signal pathway can be activated by VEGF [45, 46]. Therefore, the GBM cells were cocultured with the M2-CM, and we found that M2-CM upregulated p-PI3K, p-Akt, and Nrf2 to activate the PI3K/Akt/Nrf2 pathway, which were reversed by silencing VEGF, suggesting that M2-CM activated this pathway in a VEGF-dependent manner. Furthermore, we surprisingly found that inhibition of the PI3K/Akt/Nrf2 pathway was effective to suppress cell proliferation and stemness in the GBM cells cotreated with M2-CM, hinting that M2-CM promoted GBM aggressiveness through secreting VEGF. In addition to cancer development, the PI3K/Akt/Nrf2 pathway also participates in the regulation of chemoresistance [49, 50], and our study verified that M2-CM macrophage secreted VEGF to increase TMZ resistance in the GBM cells, which are supported by the facts that VEGF enhanced chemoresistance for multiple chemical drugs [61].

Although this study preliminarily investigated the role of M2 macrophages in accelerating cancer aggressiveness and TMZ resistance in GBM, there still existed several shortcomings needed to be resolved by the following experiments. First, this study merely investigated the peripheral macrophages in regulating GBM progression, and since the glioblastoma-associated microglia and macrophages (GAMMs) were the predominated inflammatory infiltrates during GBM progression [62, 63], the role of GAMMs in GBM development needed to be discussed in our future work. Second, hypoxic pressure changed the biological characteristics in both GBM cells and the surrounding cells [64, 65]. However, in this study, we only discussed the effects of hypoxia on M2 macrophages but not the GBM cells itself. Thus, it was still needed to be investigated whether hypoxia directly increased TMZ resistance in GBM cells. Third, the sample size of our clinical experiments was limited, and more clinical samples are needed to be collected to verify our current conclusions.

## 5. Conclusions

This study firstly investigated the underlying mechanisms by which hypoxic M2 macrophage interacted with tumor cells and HUVECs to facilitate cancer progression, TMZ resistance, and angiogenesis. The main findings of this study were summarized as follows: (1) Hypoxic stress promoted M2 polarization in TAMs through upregulating HIF-1*α*. (2) Hypoxic M2 macrophages secreted VEGF activated the PI3K/Akt/Nrf2 pathway to promote cancer aggressiveness, stemness, and TMZ resistance in GBM cells. (3) Hypoxic M2 macrophage VEGF-dependently facilitates angiogenesis in HUVECs through interacting with VEGFR.

## Data Availability

All the involved data had been included in the manuscript.

## Supplementary Materials

Supplementary Materials Figure S1: the correlations of M1/M2 macrophages with patients' (A, B) gender and (C, D) age were analyzed. Each experiment repeated at least 3 times. Figure S2: M2-CM VEGF-dependently promoted cell proliferation in the GBM cells. The shRNAs for (A) VEGF, (B) EGF, (C) PDGF, and (D) TGF-*β*1 were, respectively, transfected into the M2 macrophages, and the ELISA analysis was performed to examine their expressions in the M2-CM. (E, F) MTT assay was used to examine cell proliferation in the GBM cells. Each experiment repeated at least 3 times, and ^∗^*P* < 0.05. Figure S3: the xenograft tumor-bearing mouse models were established, and (A, B) tumor weight was obtained and weighed. Each experiment repeated at least 3 times, and ^∗^*P* < 0.05. Figure S4: the HUVECs were cocultured with macrophage-derived conditional medium, and its angiogenesis abilities were examined by performing (A, B) tube formation assay. Figure S5: immunofluorescent staining assay was performed to determine the expression status and cellular localization of Nrf2.

## References

[1] Jacob F., Salinas R. D., Zhang D. Y. (2020). A patient-derived glioblastoma organoid model and biobank recapitulates inter- and intra-tumoral heterogeneity. *Cell*.

[2] Lah T. T., Novak M., Breznik B. (2020). Brain malignancies: glioblastoma and brain metastases. *Seminars in Cancer Biology*.

[3] De Leo A., Ugolini A., Veglia F. (2021). Myeloid cells in glioblastoma microenvironment. *Cells*.

[4] DeCordova S., Shastri A., Tsolaki A. G. (2020). Molecular heterogeneity and immunosuppressive microenvironment in glioblastoma. *Frontiers in Immunology*.

[5] Li M., Kirtane A. R., Kiyokawa J. (2020). Local targeting of NAD(+) salvage pathway alters the immune tumor microenvironment and enhances checkpoint immunotherapy in glioblastoma. *Cancer Research*.

[6] Zhu Z., Zhang H., Chen B. (2020). PD-L1-mediated immunosuppression in glioblastoma is associated with the infiltration and M2-polarization of tumor-associated macrophages. *Frontiers in Immunology*.

[7] Akkari L., Bowman R. L., Tessier J. (2020). Dynamic changes in glioma macrophage populations after radiotherapy reveal CSF-1R inhibition as a strategy to overcome resistance. *Science Translational Medicine*.

[8] Sa J. K., Chang N., Lee H. W. (2020). Transcriptional regulatory networks of tumor-associated macrophages that drive malignancy in mesenchymal glioblastoma. *Genome Biology*.

[9] Yin J., Kim S. S., Choi E. (2020). ARS2/MAGL signaling in glioblastoma stem cells promotes self-renewal and M2-like polarization of tumor-associated macrophages. *Nature Communications*.

[10] Shi Y., Zhang B., Zhu J. (2020). miR-106b-5p inhibits IRF1/IFN-*β* signaling to promote M2 macrophage polarization of glioblastoma. *Oncotargets and Therapy*.

[11] Tao W., Chu C., Zhou W. (2020). Yu X and others. Dual role of WISP1 in maintaining glioma stem cells and tumor-supportive macrophages in glioblastoma. *Nature Communications*.

[12] Yang Y. P., Chien C. S., Yarmishyn A. A. (2021). Musashi-1 regulates MIF1-mediated M2 macrophage polarization in promoting glioblastoma progression. *Cancers*.

[13] White M. J. V., Briquez P. S., White D. A. V., Hubbell J. A. (2021). VEGF-A, PDGF-BB and HB-EGF engineered for promiscuous super affinity to the extracellular matrix improve wound healing in a model of type 1 diabetes. *NPJ Regenerative Medicine*.

[14] Zhang J., Li H., Wu Q. (2019). Tumoral NOX4 recruits M2 tumor-associated macrophages via ROS/PI3K signaling-dependent various cytokine production to promote NSCLC growth. *Redox Biology*.

[15] Gomez-Roman N., Chong M. Y., Chahal S. K. (2020). Radiation responses of 2D and 3D glioblastoma cells: a novel, 3D-specific radioprotective role of VEGF/Akt signaling through functional activation of NHEJ. *Molecular Cancer Therapeutics*.

[16] Nicolas S., Abdellatef S., Haddad M. A., Fakhoury I., El-Sibai M. (2019). Hypoxia and EGF stimulation regulate VEGF expression in human glioblastoma multiforme (GBM) cells by differential regulation of the PI3K/Rho-GTPase and MAPK pathways. *Cell*.

[17] Lu C., Wei Y., Wang X. (2020). DNA-methylation-mediated activating of lncRNA SNHG12 promotes temozolomide resistance in glioblastoma. *Molecular Cancer*.

[18] Wei Y., Lu C., Zhou P. (2021). EIF4A3-induced circular RNA ASAP1 promotes tumorigenesis and temozolomide resistance of glioblastoma via NRAS/MEK1/ERK1-2 signaling. *Neuro-Oncology*.

[19] Noh K. H., Lee S. H., Lee H. (2022). Novel cancer stem cell marker MVP enhances temozolomide-resistance in glioblastoma. *Translational Oncology*.

[20] Yang W. B., Hsu C. C., Hsu T. I. (2020). Increased activation of HDAC1/2/6 and Sp1 underlies therapeutic resistance and tumor growth in glioblastoma. *Neuro-Oncology*.

[21] Miyazaki T., Ishikawa E., Matsuda M. (2020). Infiltration of CD163-positive macrophages in glioma tissues after treatment with anti-PD-L1 antibody and role of PI3K*γ* inhibitor as a combination therapy with anti-PD-L1 antibody in in vivo model using temozolomide-resistant murine glioma-initiating cells. *Brain Tumor Pathology*.

[22] Chuang H. Y., Su Y. K., Liu H. W. (2019). Preclinical evidence of STAT3 inhibitor pacritinib overcoming temozolomide resistance via downregulating miR-21-enriched exosomes from M2 glioblastoma-associated macrophages. *Journal of Clinical Medicine*.

[23] Chang J., Li H., Zhu Z. (2022). microRNA-21-5p from M2 macrophage-derived extracellular vesicles promotes the differentiation and activity of pancreatic cancer stem cells by mediating KLF3. *Cell Biology and Toxicology*.

[24] Lv J., Liu C., Chen F. K. (2021). M2-like tumour-associated macrophage-secreted IGF promotes thyroid cancer stemness and metastasis by activating the PI3K/AKT/mTOR pathway. *Molecular Medicine Reports*.

[25] Colwell N., Larion M., Giles A. J. (2017). Hypoxia in the glioblastoma microenvironment: shaping the phenotype of cancer stem-like cells. *Neuro-Oncology*.

[26] Jawhari S., Ratinaud M. H., Verdier M. (2016). Glioblastoma, hypoxia and autophagy: a survival-prone 'menage-a-trois'. *Cell Death & Disease*.

[27] Park J. E., Dutta B., Tse S. W. (2019). Hypoxia-induced tumor exosomes promote M2-like macrophage polarization of infiltrating myeloid cells and micro RNA-mediated metabolic shift. *Oncogene*.

[28] Qi L., Chen J., Yang Y., Hu W. (2020). Hypoxia correlates with poor survival and M2 macrophage infiltration in colorectal cancer. *Frontiers in Oncology*.

[29] Lo Dico A., Martelli C., Diceglie C., Lucignani G., Ottobrini L. (2018). Hypoxia-inducible factor-1*α* activity as a switch for glioblastoma responsiveness to temozolomide. *Frontiers in Oncology*.

[30] Boyd N. H., Tran A. N., Bernstock J. D. (2021). Glioma stem cells and their roles within the hypoxic tumor microenvironment. *Theranostics*.

[31] Cristofaro I., Limongi C., Piscopo P. (2020). M2 receptor activation counteracts the glioblastoma cancer stem cell response to hypoxia condition. *International Journal of Molecular Sciences*.

[32] Ahir B. K., Engelhard H. H., Lakka S. S. (2020). Tumor development and angiogenesis in adult brain tumor: glioblastoma. *Molecular Neurobiology*.

[33] Fu Y., Wang D., Wang H. (2020). TSPO deficiency induces mitochondrial dysfunction, leading to hypoxia, angiogenesis, and a growth-promoting metabolic shift toward glycolysis in glioblastoma. *Neuro-Oncology*.

[34] Zhu Y., Liu X., Zhao P., Zhao H., Gao W., Wang L. (2020). Celastrol suppresses glioma vasculogenic mimicry formation and angiogenesis by blocking the PI3K/Akt/mTOR signaling pathway. *Frontiers in Pharmacology*.

[35] Dong F., Ruan S., Wang J. (2020). M2 macrophage-induced lncRNA PCAT6 facilitates tumorigenesis and angiogenesis of triple-negative breast cancer through modulation of VEGFR2. *Cell Death & Disease*.

[36] Yang Y., Guo Z., Chen W. (2021). Wu W and others. M2 macrophage-derived exosomes promote angiogenesis and growth of pancreatic ductal adenocarcinoma by targeting E2F2. *Molecular Therapy*.

[37] Kao J. K., Wang S. C., Ho L. W. (2020). M2-like polarization of THP-1 monocyte-derived macrophages under chronic iron overload. *Annals of Hematology*.

[38] Okamoto Y., Kitakaze K., Takenouchi Y., Yamamoto S., Ishimaru H., Tsuboi K. (2021). Sphingosine 1-phosphate receptor type 2 positively regulates interleukin (IL)-4/IL-13-induced STAT6 phosphorylation. *Cellular Signalling*.

[39] Pinto S. M., Kim H., Subbannayya Y. (2021). Comparative proteomic analysis reveals varying impact on immune responses in phorbol 12-myristate-13-acetate-mediated THP-1 monocyte-to-macrophage differentiation. *Frontiers in Immunology*.

[40] Jiang Q., Geng X., Warren J. (2020). Hypoxia inducible factor-1*α* (HIF-1*α*) mediates NLRP3 inflammasome-dependent-pyroptotic and apoptotic cell death following ischemic stroke. *Neuroscience*.

[41] Liu J., Qiu P., Qin J. (2020). Allogeneic adipose-derived stem cells promote ischemic muscle repair by inducing M2 macrophage polarization via the HIF-1*α*/IL-10 pathway. *Stem Cells*.

[42] Sokulsky L. A., Goggins B., Sherwin S. (2020). GSTO1-1 is an upstream suppressor of M2 macrophage skewing and HIF-1*α*-induced eosinophilic airway inflammation. *Clinical and Experimental Allergy*.

[43] Long Y., Tao H., Karachi A. (2020). Dysregulation of glutamate transport enhances Treg function that promotes VEGF blockade resistance in glioblastoma. *Cancer Research*.

[44] Patel K. S., Yao J., Raymond C. (2020). Oughourlian T and others. Decorin expression is associated with predictive diffusion MR phenotypes of anti-VEGF efficacy in glioblastoma. *Scientific Reports*.

[45] Wen N., Guo B., Zheng H. (2019). Bromodomain inhibitor jq1 induces cell cycle arrest and apoptosis of glioma stem cells through the VEGF/PI3K/AKT signaling pathway. *International Journal of Oncology*.

[46] Zhang W., Wu Y., Chen H., Yu D., Zhao J., Chen J. (2021). Neuroprotective effects of SOX5 against ischemic stroke by regulating VEGF/PI3K/AKT pathway. *Gene*.

[47] Wang H., Zhou X. M., Wu L. Y. (2020). Aucubin alleviates oxidative stress and inflammation via Nrf 2-mediated signaling activity in experimental traumatic brain injury. *Journal of Neuroinflammation*.

[48] Wei J., Wang Z., Wang W. (2021). Oxidative stress activated by sorafenib alters the temozolomide sensitivity of human glioma cells through autophagy and JAK2/STAT3-AIF axis. *Frontiers in Cell and Development Biology*.

[49] Gao A. M., Ke Z. P., Shi F., Sun G. C., Chen H. (2013). Chrysin enhances sensitivity of BEL-7402/ADM cells to doxorubicin by suppressing PI3K/Akt/Nrf2 and ERK/Nrf2 pathway. *Chemico-Biological Interactions*.

[50] Gao A. M., Ke Z. P., Wang J. N., Yang J. Y., Chen S. Y., Chen H. (2013). Apigenin sensitizes doxorubicin-resistant hepatocellular carcinoma BEL-7402/ADM cells to doxorubicin via inhibiting PI3K/Akt/Nrf2 pathway. *Carcinogenesis*.

[51] Lan J., Sun L., Xu F. (2019). M2 macrophage-derived exosomes promote cell migration and invasion in colon cancer. *Cancer Research*.

[52] Weng Y. S., Tseng H. Y., Chen Y. A. (2019). MCT-1/miR-34a/IL-6/IL-6R signaling axis promotes EMT progression, cancer stemness and M2 macrophage polarization in triple-negative breast cancer. *Molecular Cancer*.

[53] Zhao S., Mi Y., Guan B. (2020). Tumor-derived exosomal miR-934 induces macrophage M2 polarization to promote liver metastasis of colorectal cancer. *Journal of Hematology & Oncology*.

[54] Huang B. R., Liu Y. S., Lai S. W. (2020). CAIX regulates GBM motility and TAM adhesion and polarization through EGFR/STAT3 under hypoxic conditions. *International Journal of Molecular Sciences*.

[55] Shan T., Chen S., Chen X. (2020). M2-TAM subsets altered by lactic acid promote T-cell apoptosis through the PD-L1/PD-1 pathway. *Oncology Reports*.

[56] Yang X., Liu H., Ye T. (2020). AhR activation attenuates calcium oxalate nephrocalcinosis by diminishing M1 macrophage polarization and promoting M2 macrophage polarization. *Theranostics*.

[57] Karami A., Hossienpour M., Mohammadi Noori E., Rahpyma M., Najafi K., Kiani A. (2022). Synergistic effect of gefitinib and temozolomide on U87MG glioblastoma angiogenesis. *Nutrition and Cancer*.

[58] Llaguno-Munive M., León-Zetina S., Vazquez-Lopez I., Ramos-Godinez M. D. P., Medina L. A., Garcia-Lopez P. (2020). Mifepristone as a potential therapy to reduce angiogenesis and P-glycoprotein associated with glioblastoma resistance to temozolomide. *Frontiers in Oncology*.

[59] Shahcheraghi S. H., Tchokonte-Nana V., Lotfi M., Lotfi M., Ghorbani A., Sadeghnia H. R. (2020). Wnt/beta-catenin and PI3K/Akt/mTOR signaling pathways in glioblastoma: two main targets for drug design: a review. *Current Pharmaceutical Design*.

[60] Zhou Y., An H., Wu G. (2020). MicroRNA-6071 suppresses glioblastoma progression through the inhibition of PI3K/AKT/mTOR pathway by binding to ULBP2. *Oncotargets and Therapy*.

[61] Liu P., Li X., Cui Y. (2019). LncRNA-MALAT1 mediates cisplatin resistance via miR-101-3p/VEGF-C pathway in bladder cancer. *Acta Biochimica et Biophysica Sinica*.

[62] Poon C. C., Sarkar S., Yong V. W., Kelly J. J. P. (2017). Glioblastoma-associated microglia and macrophages: targets for therapies to improve prognosis. *Brain*.

[63] Xuan W., Lesniak M. S., James C. D., Heimberger A. B., Chen P. (2021). Context-dependent glioblastoma-macrophage/microglia symbiosis and associated mechanisms. *Trends in Immunology*.

[64] Takkar S., Sharma V., Ghosh S., Suri A., Sarkar C., Kulshreshtha R. (2021). Hypoxia-inducible miR-196a modulates glioblastoma cell proliferation and migration through complex regulation of NRAS. *Cellular Oncology (Dordrecht)*.

[65] Zhang B., Chen Y., Shi X. (2021). Regulation of branched-chain amino acid metabolism by hypoxia-inducible factor in glioblastoma. *Cellular and Molecular Life Sciences*.

