# Supplementary material for: Hypoxia-Driven M2-Polarized Macrophages Facilitate Cancer Aggressiveness and Temozolomide Resistance in Glioblastoma
Source: Oxid Med Cell Longev. 2022 Aug 22;2022:1614336. doi: 10.1155/2022/1614336 (PMC9423979; doi:10.1155/2022/1614336)
Supplement: Supplementary Materials — Figure S1: the correlations of M1/M2 macrophages with patients' (A, B) gender and (C, D) age were analyzed. Each experiment repeated at least 3 times. Figure S2: M2-CM VEGF-dependently promoted cell proliferation in the GBM cells. The shRNAs for (A) VEGF, (B) EGF, (C) PDGF, and (D) TGF-β1 were, respectively, transfected into the M2 macrophages, and the ELISA analysis was performed to examine their expressions in the M2-CM. (E, F) MTT assay was used to examine cell proliferation in the GBM cells. Each experiment repeated at least 3 times, and ∗P < 0.05. Figure S3: the xenograft tumor-bearing mouse models were established, and (A, B) tumor weight was obtained and weighed. Each experiment repeated at least 3 times, and ∗P < 0.05. Figure S4: the HUVECs were cocultured with macrophage-derived conditional medium, and its angiogenesis abilities were examined by performing (A, B) tube formation assay. Figure S5: immunofluorescent staining assay was performed to determine the expression status and cellular localization of Nrf2. [file 1614336.f1.docx]

**Supplementary Figure legends**


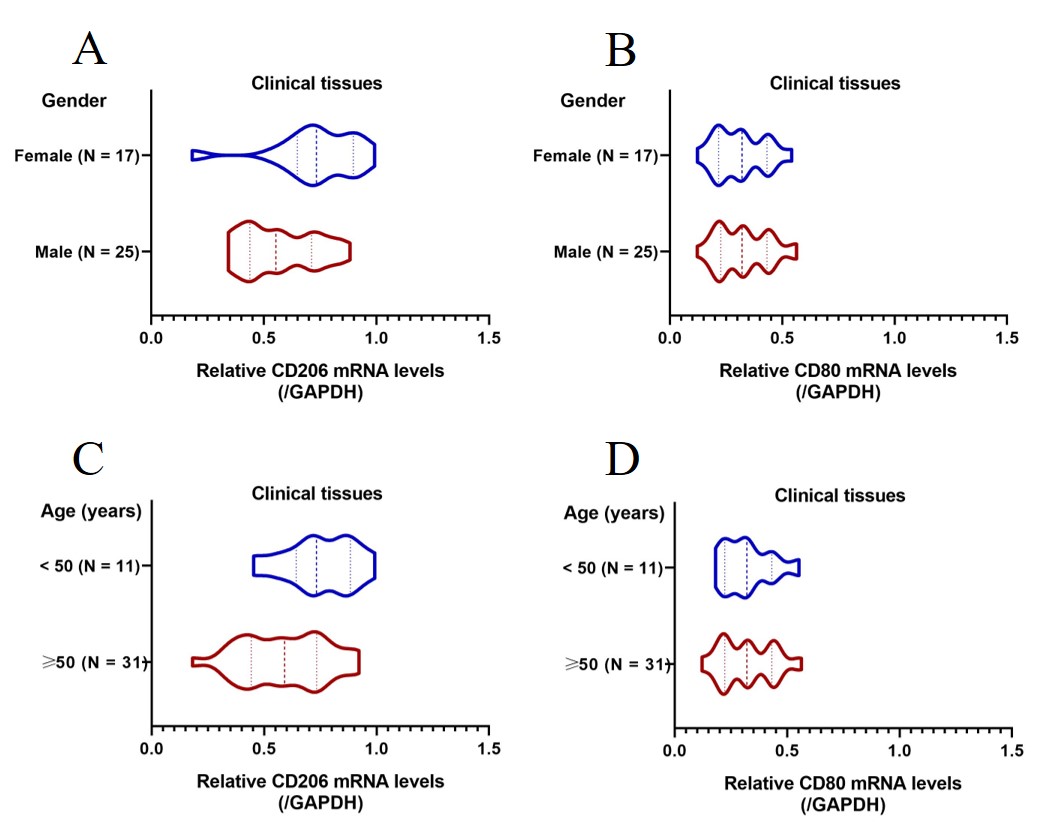


**Figure S1.** The correlations of M1/M2 macrophages with patients’ (A, B) gender and (C, D) age were analyzed. Each experiment repeated at least 3 times.


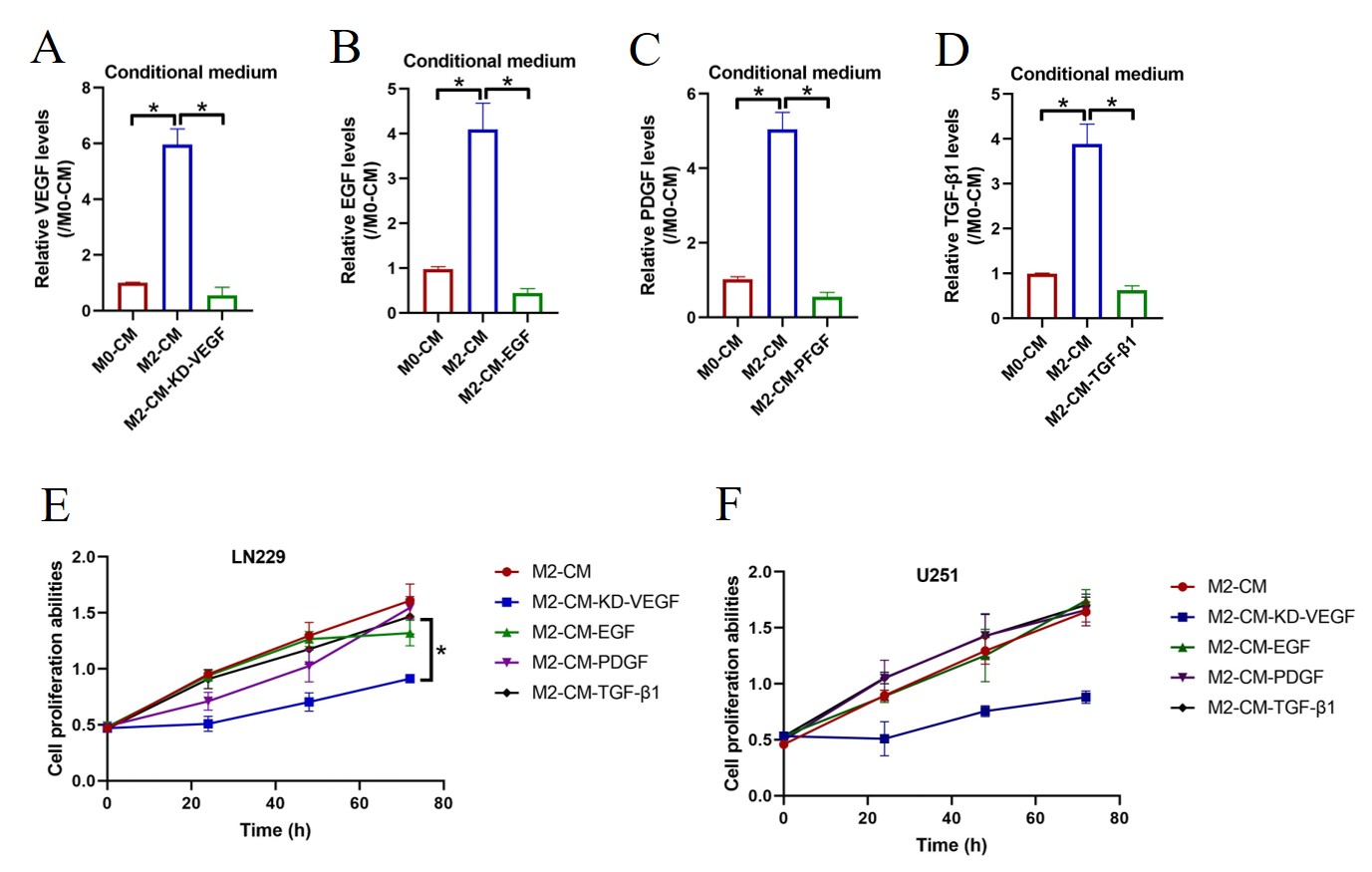


**Figure S2.** M2-CM VEGF-dependently promoted cell proliferation in the GBM cells. The shRNAs for (A) VEGF, (B) EGF, (C) PDGF, (D) TGF-β1 were respectively transfected into the M2 macrophages, and the ELISA analysis was performed to examine their expressions in the M2-CM. (E, F) MTT assay was used to examine cell proliferation in the GBM cells. Each experiment repeated at least 3 times, and **P* < 0.05.


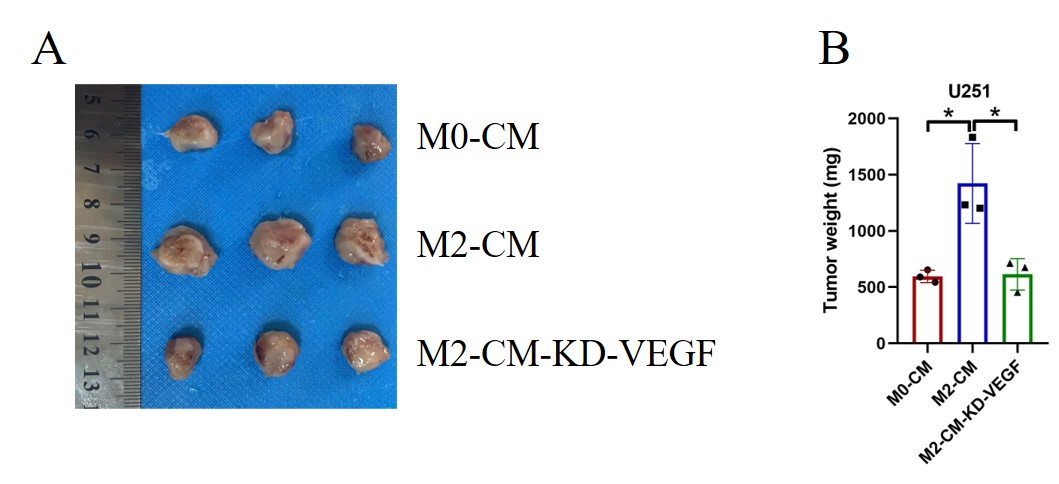


**Figure S3.** The xenograft tumor-bearing mice models were established, and (A, B) tumor weight was obtained and weighed. Each experiment repeated at least 3 times, and **P* < 0.05.


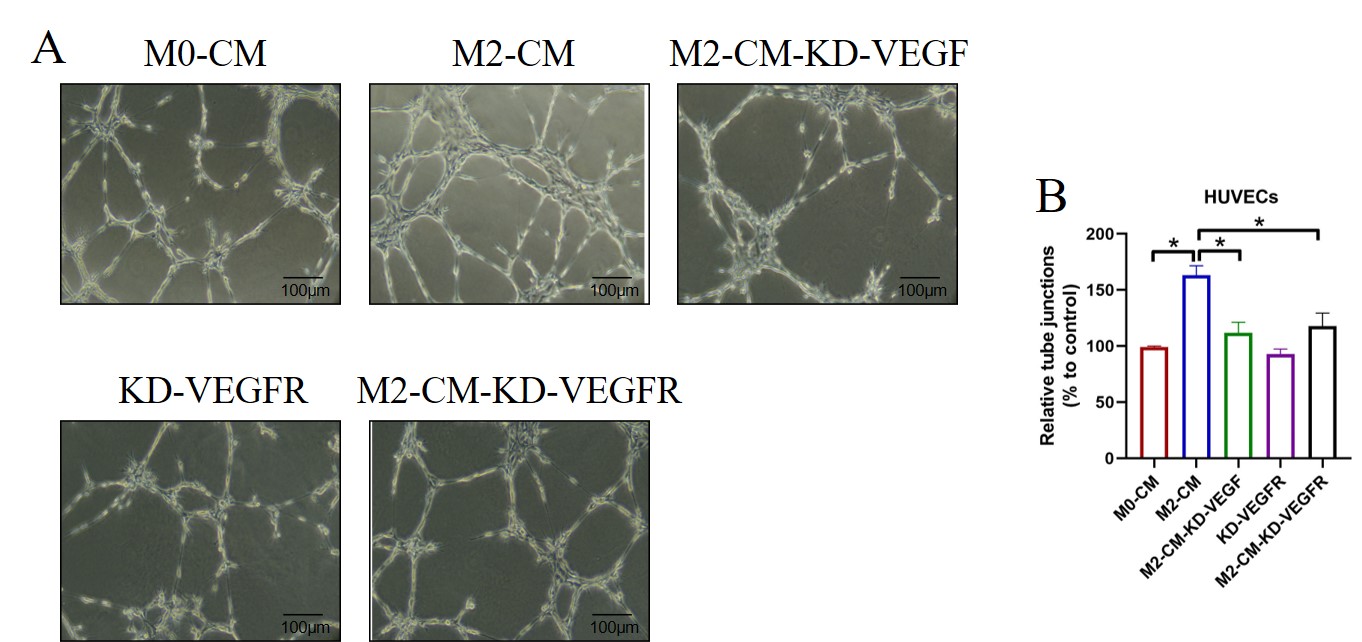


**Figure S4.** The HUVECs were co-cultured with macrophages-derived conditional medium, and its angiogenesis abilities were examined by performing (A, B) tube formation assay.


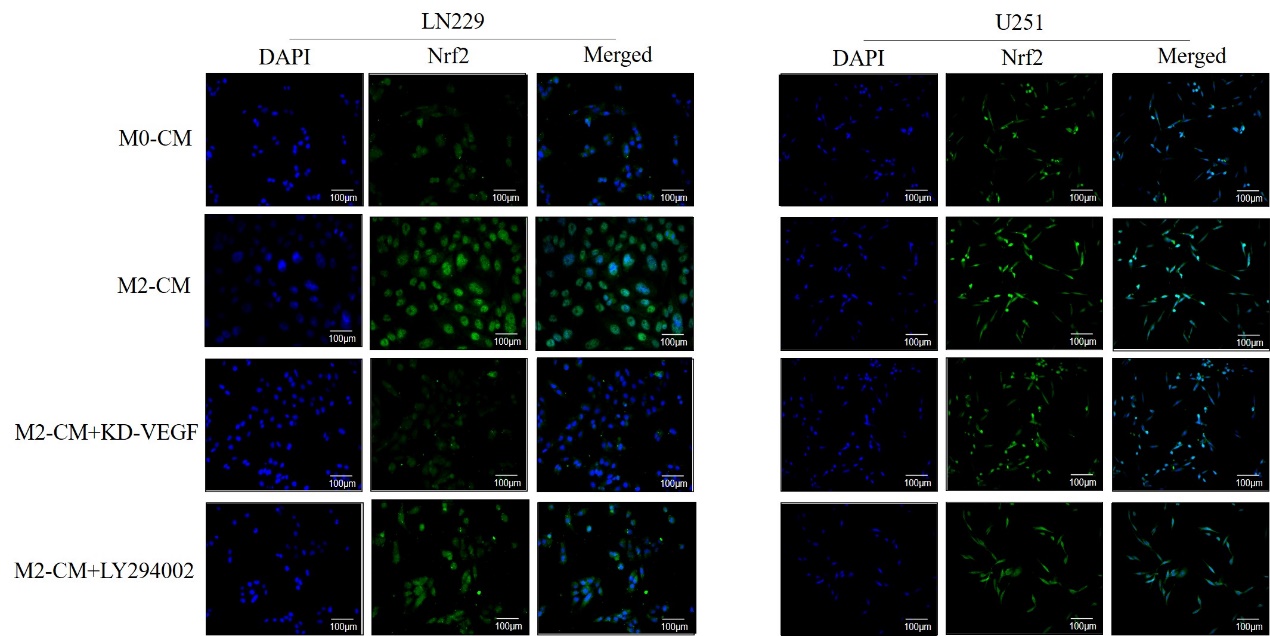


**Figure S5.** Immunofluorescent staining assay was performed to determine the expression status and cellular localization of Nrf2.
